# Workplace Violence against Health Care Workers in North Chinese Hospitals: A Cross-Sectional Survey

**DOI:** 10.3390/ijerph14010096

**Published:** 2017-01-19

**Authors:** Peihang Sun, Xue Zhang, Yihua Sun, Hongkun Ma, Mingli Jiao, Kai Xing, Zheng Kang, Ning Ning, Yapeng Fu, Qunhong Wu, Mei Yin

**Affiliations:** 1 Department of Health, Policy and Hospital Management, School of Public Health, Harbin Medical University, Harbin 150081, China; sun745450570@163.com (P.S.); xingkai19921202@163.com (K.X.); 2 School of Humanities and Social Sciences, Harbin Medical University, Harbin 150081, China; xuezhang1212@sina.com; 3 Human Resources Department, Shanghai Mental Health Center, Shanghai 200000, China; yihuasunS@163.com; 4 Department of Finance and Public Management, Harbin University of Commerce, Harbin 150081, China; hongkunmaM@163.com; 5 Institute of Quantitative and Technical Economics, Chinese Academy of Social Science, Beijing 100000, China; 6 Department of Social Medicine, School of Public Health, Harbin Medical University, Harbin 150081, China; zhengkang1983@126.com (Z.K.); hyhyjw@126.com (N.N.); 7 Graduate Department of Cancer Hospital Affiliated to Harbin Medical University, Harbin 150000, China; yapengfu@163.com

**Keywords:** workplace violence, risk factors, different types of violence

## Abstract

This research aimed to determine the prevalence of workplace violence (WPV) against healthcare workers, explore the frequency distribution of violence in different occupational groups, and determine which healthcare occupation suffers from WPV most frequently. Furthermore, the current study aimed to compare risk factors affecting different types of WPV in Chinese hospitals. A cross-sectional design was utilized. A total of 1899 healthcare workers from Heilongjiang, a province in Northeastern China, completed the questionnaire. Of the respondents, 83.3% reported exposure to workplace violence, and 68.9% reported non-physical violence. Gender, education, shift work, anxiety level, and occupation were significantly correlated with physical violence (*p* < 0.05 for all correlations). Additionally, age, professional title, and occupation were correlated with non-physical violence, which critically affected doctors. Thus, gender, age, profession, anxiety, and shift work were predictive of workplace violence toward healthcare workers. Doctors appeared to experience non-physical workplace violence with particularly higher frequency when compared to nurses and other workers in hospitals. For healthcare workers, interventions aimed at WPV reduction should be enacted according to the types of violence, profession, and other factors underlying the various types of WPV in hospitals.

## 1. Introduction

Prevention and control of occupational injuries is one of the most important priorities in the field of public health [1]. Hospitals have a special status and role in public health and healthcare, as they constitute the terminal point of health service systems. The National Institute for Occupational Safety and Health (NIOSH) notes that the public place where most violence toward employees can be observed is hospitals [2]. In other words, health care workers are the professionals most vulnerable to workplace violence (WPV) [3].

WPV results in great harm. From a microscopic view, WPV can affect the physical and mental health of victims [3]. As such, violence against healthcare workers is a major problem affecting health and productivity. Additionally, WPV can directly cause short- or long-term absenteeism and degrade the work climate and morale of healthcare workers [4]. Further, from a macro point of view, WPV in the health sector can significantly affect the effectiveness healthcare systems, particularly in developing countries [5].

In China, the medical and health service system is still developing [6,7]. Additionally, technical services and communication in medicine are less complete and less proactively implemented than in fully developed countries. At present, medical relations are highly volatile, and negative media coverage has adversely affected the public credibility of hospitals, resulting in doctors fearing patients and patients no longer trusting doctors [8]. Within this context, reports of violent attacks on healthcare workers have increased, including some fatal injuries. For example, in 2012, at the First Affiliated Hospital of Harbin Medical University (a hospital sampled in this study), a 17-year-old boy with ankylosing spondylitis and tuberculosis fatally stabbed Wang Hao, a young intern who had accepted an offer to enrol in Hong Kong University’s Ph.D. program, and was leaving the ward. Unfortunately [9], this incident is not an anomaly, as abuse of healthcare workers is common [10]. For instance, on 25 October 2013, in an elevator of Wenling First Hospital in Zhejiang province, Dr Shan, a thoracic surgeon, was assaulted by the relative of a patient. Later, the perpetrator stated, “I just like to attack any doctor in a white lab coat for no reason” [11]. In a collection of 124 detailed media reports of incidents involving serious violence in hospitals gathered over the 10 years prior to 2011, 29 healthcare workers were murdered, and 52 seriously injured. Most victims were doctors and nurses, including a doctor who had acid poured on his face, another who had her throat cut, and an explosion in a hospital that caused five deaths and several injuries [12]. Naturally, other forms of violence also occur relatively frequently. For instance, among 5061 medical staff in 17 hospitals in Guangdong Province, the incidence of violence was 58.23%, within which the incidence rates of psychological, physical, and sexual violence were 56.85%, 12.85%, and 6.99%, respectively [13].

These increasingly frequent violent incidents have aroused the attention of researchers all over the world. Researchers working in developed countries have measured WPV among healthcare workers, and prevention and control strategies have been recommended and implemented to control WPV [5]. However, this topic has rarely been addressed in developing or low-income countries. In studies examining the victims of violence, specifically in hospitals outside of China, research suggests that nurses experience the highest levels of violence, which may be related to increased contact with patients.

There is no single uniform definition or categorization of workplace violence (WPV). This paper utilizes the definition of WPV put forth by the World Health Organization (WHO); specifically, the intentional use of physical force or power, threatened or actual, against oneself, another person, group, or community, that either results in, or has a high likelihood of resulting in, injury, death, and/or psychological or developmental harm or deprivation [3]. Following the WHO’s definition, workplace violence takes two main forms: physical and non-physical violence. Physical violence includes hitting, slapping, kicking, pushing, choking, grabbing, sexual assault, and other forms of physical contact intended to injure or harm. In contrast, non-physical violence includes threats, sexual harassment, bullying, and verbal abuse and may be perpetrated by various types of people [14].

As such, an increasing amount of research has conducted in-depth studies with nurse populations, including an examination of WPV frequency, causes, influencing factors, and preventive measures. In studies of the impact of WPV on the whole medical staff, contradictory research findings indicate that males experience significantly more physical violence than females, and that gender is not associated with either physical or psychological violence [5,15]. WPV exposure is significantly related to anxiety in healthcare workers [16]. In Taiwan, above-moderate anxiety was associated with an increased likelihood of physical and verbal violence, after controlling for other risk factors, and health staff aged ≤44 years were more likely to report physical violence than older staff members [16]. Among health professionals, doctors, nurses, and ambulance staff reported the highest prevalence of physical violence [15]. In hospitals, shift work does not appear to significantly affect rates of reported physical or non-physical violent incidents [5].

Several studies have examined WPV risk factors in hospitals and among doctors and nurses [16,17,18]. However, WPV victims in hospitals are not confined to these groups. Other medical, administrative, and miscellaneous positions, in indirect contact with clients, may experience WPV [19]. Therefore, in this study, we expanded the range of individuals examined regarding exposure to WPV.

In recent years, public and media reports addressing WPV in Chinese healthcare systems have typically used the expression “medical violent injury.” On the surface, use of this description makes violence in hospitals appear to mainly affect doctors rather than nurses [20]. The roles and positioning of doctors and nurses are not the same in the Chinese medical and health system, when compared to other developed countries. For instance, a particular phenomenon exists in some Chinese hospitals, as family members of patients carry out basic nursing work over the long term, and doctors carry out advanced nursing procedures [3], resulting in doctors coming into more contact with patients. Given this information, it is questionable as to whether nurses in China would be the group most affected by WPV, in the same manner as other developed countries. Furthermore, WPV in hospitals differs depending on particular contexts within the hospital (i.e., the type of WPV). Therefore, it is reasonable to assume that the factors affecting WPV in hospitals will differ depending on the particular type of WPV [21].

Based on this, the current study aimed to determine the prevalence of WPV against healthcare workers, explore the frequency distribution of violence in different professional groups, and determine which healthcare profession suffers from WPV most frequently in Chinese hospitals, in an effort to develop better interventions and preventive measures. Additionally, the current study sought to compare risk factors affecting different types of WPV.

## 2. Materials and Methods

The hospitals examined in this research were located in the Heilongjiang province in Northeastern China, which has a population of 38.1 million people, and contains 69 tertiary hospitals. Due to time and resource limitations, seven hospitals were deliberately selected to represent various areas of the province (east, central, and west), and all seven hospitals responded. Harbin, the capital city of the province, contains the most concentrated collection of tertiary hospitals. We therefore selected four hospitals from Harbin (in the central area of the province). Additionally, we selected two hospitals from Qigihar (the western part of the province) and one hospital from Jiamusi (the eastern part of the province).

### 2.1. Data Collection

A non-probabilistic purposive strategy was used select 400 healthcare workers from each of the chosen hospitals. We first obtained lists of all healthcare workers employed at each hospital from hospital managers and human resource personnel. We combined these lists, assigned a number to the name of each worker, and selected an average of 400 personnel from each hospital. These samples included doctors, nurses, and members of various other professions (e.g., medical technicians and administrative staff). Before distributing the questionnaire, all respondents were well informed of the purposes and methods of the study in a notification letter.

After voluntarily consenting to participate, each respondent completed and returned an anonymous questionnaire to a box provided in the office of the manager. Respondent names and other identifiers were not required. Using this procedure, we collected 1899 valid responses (valid response rate: 67.8%). Data were collected between July 2013 and September 2014.

### 2.2. Questionnaire

The questionnaire we used to measure WPV was developed in 2003 by an International Labour Office (ILO), International Council of Nurses (ICN), WHO, and Public Services International (PSI) joint program to measure workplace violence [18]. First, we formally obtained documented permission to use the questionnaire from the ILO and WHO. The questionnaire was then translated into Mandarin Chinese and back-translated into English to verify the accuracy of the Mandarin version. Subsequently, we invited 17 healthcare-related experts from all over China to evaluate the content validity of the measure, including suitability for Chinese culture, and the appropriateness of the translation. The two-week test–retest reliability was supported using a group of 37 healthcare workers at five of the surveyed hospitals (Cronbach’s α = 0.87).

The questionnaire had the following sections. The first section collected demographic and workplace data (i.e., age, sex, years of experience, marital status, level of education, rank or title, profession, participation in shift work), departmental data, and a question about anxiety regarding WPV, which was measured on a scale from never to extremely high. The second section examined respondent experiences with physical violence in the past 12 months. The third section examined respondent experiences with non-physical violence (i.e., verbal abuse, threatening events, and sexual harassment) in the past 12 months. The fourth section contained three open-ended questions examining respondent opinions towards WPV.

### 2.3. Data Analysis

Descriptive statistics were calculated for demographic characteristics and frequency of experience with WPV. Frequency distributions were calculated (in percentage points) for physical and non-physical violence experienced by personnel in each occupational category. A chi-square test was performed as a relevance analysis of suffering physical violence and respondent characteristics. Additionally, logistic regression analysis was used to determine which demographic characteristics (including gender, marital status, age, education level, professional title, whether participants worked night shifts, occupation, and anxiety level) predicted physical and non-physical violence. SPSS v.19.0 was used for all analysis (Statistical Product and Service Solutions 19.0, 2010, IBM Corporation, Armonk, NY, USA). Values of *p* < 0.05 were considered significant.

### 2.4. Ethical Considerations

Ethical approval was granted by the Institutional Review Board of Harbin Medical University before data collection commenced (Project Identification Code: HMUIRB20160014). All of the study procedures were approved by each study hospital, and all of the participants provided their informed consent to participate.

## 3. Results

### 3.1. Demographic Characteristics of Respondents

These are summarized in Table 1.

The most typical respondents were female (60.9%), aged 30–39 years (40.3%), held an undergraduate degree or higher (67.9%), and shift work (70.9%). The primary department in which respondents regularly worked was internal medicine (37.1%), followed by surgery departmental work (33.4%). More than half of respondents who had experienced WPV were doctors (57.2%), and 39.1% of respondents reported extreme anxiety regarding WPV (Table 1).

### 3.2. Violence by Occupational Category

A total of 83.3% of respondents reported exposure to workplace violence; 14.4% and 68.9% reported exposure to physical and non-physical violence, respectively. Affected personnel included doctors, nurses, and technical, administrative, and rear-end service personnel. Doctors and nurses primarily reported exposure to physical and non-physical violence. These are shown in Table 2.

### 3.3. Characteristics and Frequency Distributions for Physical and Non-Physical Violence

Gender, education level, shift work, and anxiety were significantly correlated with physical violence (*p* < 0.05 for all). Respondents reporting extreme anxiety most frequently reported having experienced WPV (23.6%, *n* = 175), followed by graduate degree holders (21.2%, *n* = 70), and males (19.3%, *n* = 143). Regarding non-doctors, we found that males faced the greatest risk. In addition, age, education level, professional title, shift work, occupation, and anxiety level correlated with respondent experience of non-physical violence. These are shown in Table 3.

### 3.4. Multiple Logistic Regression of Physical and Non-Physical Violence

Table 4 presents the results of multiple logistic regression of physical and non-physical WPV, including 95% confidence intervals, and according to respondent characteristics. Among them, respondents who felt extreme anxiety regarding physical WPV were most likely to have experienced both physical and non-physical WPV, followed by individuals who reported high anxiety in non-physical violence. Respondents who participated in shift work were 1.9 times more likely to have experienced WPV than respondents who did not (*p* < 0.05). In addition, it can be seen that doctors were the most vulnerable to violence of a non-physical nature, followed by nurses, medical technicians, and administrators.

## 4. Discussion

Shift work is common in general hospitals in China and typically involves night shift duty [22]. In this study, workers who participated in shift work were 1.91 and 1.89 times more likely to experience physical and non-physical violence, respectively, compared with workers who worked only during the day. In contrast, a study in Palestine found no significant correlation between exposure to WPV and shift work [5]. Timely breaks mitigate the effects of employee fatigue and improve alertness; however, shift work consistently appears to increase pathological fatigue [23], resulting in some form of physical impact. In medicine and epidemiology, shift work is considered as a risk factor for some types of personal health problems, for example, the pathological fatigue. This is because working in shifts disrupts the circadian rhythm, which typically increases the development of physical illnesses, and brings about fatigue [24].

Higher anxiety about WPV typically is associated with a greater risk of exposure to WPV [25]. As such, the present results support this relationship. Furthermore, previous studies have shown that the relationship between stress and violence is cyclical. Work-related stress increases the probability of being a victim of violence, and people exposed to workplace violence are typically distressed [26]. Whittington’s and Wykes’ periodic cycle models indicate that pressure caused by exposure to violence led to impaired staff performance and adoption of behaviours which in turn increases the likelihood of recurrence of violence [27]. Future research should further examine this relationship and its underlying factors.

In this study, males were significantly more likely to have experienced physical violence, supporting previous research [28,29]. Conversely, greater age has been shown to protect against WPV in general hospitals [28], which was also supported in the current study. A possible explanation for this relationship is that older workers generally have a higher rank than younger employees, and this may reduce direct contact with patients and consequently violence [30].

In research conducted in Saudi Arabia, nurses were significantly more exposed to WPV than doctors [31]. The present study differentiated between physical and non-physical violence, and healthcare professionals experienced these types of WPV at different rates. Specifically, doctors were 1.61 times more likely than administrative staff to report having experienced non-physical WPV, followed by nurses, medical technicians, and administrative staff. An important factor in the genesis of aggression is the perpetrator and victim being alone (i.e., out of the sight of others that may intervene). It was reported that doctors can be attacked more frequently than nurses in hospitals. This may be due to the fact that doctors often work alone, while nurses work in groups [32].

Medical workers in China face stresses due to insufficient medical staff. In other countries, nurses have certain independent rights of prescription. In contrast, a particular phenomenon exists in some Chinese hospitals, where family members of patients carry out basic nursing work over the long term, and doctors carry out advanced nursing. This has seriously increased the stress affecting doctors in China. In 2013, 2,795,000 doctors were practicing in China, resulting in a ratio of around 1.5 doctors per 1000 persons; this ratio is around half of Brazil. Nonetheless, large cities contain 80% of the available medical resources, of which 30% are concentrated in large hospitals. Additionally, doctors in large general hospitals typically work nearly 10 h of overtime per week, which is extremely unusual among developed countries. Consequently, long-term overwork likely increases the risk of exposure to WPV in doctors. This is because different occupational orientation provides more contact opportunities with patients. This heavy load of patient visits increases work pressure in doctors, resulting in long-term overwork. As a result, this will greatly increase the likelihood of doctors being exposed to hospital violence.

## 5. Limitations

This study has the following limitations. First, hospitals in only one province were surveyed. Furthermore, a third of the sample was derived from surgical employees. Therefore, the present results may not generalize to other contexts. Moreover, because of the time and resource restrictions, our study was limited to select purposive sampling rather than quota sampling. Additionally, responses would inevitably have been affected by recall bias, as respondents were required to recall events from the past 12 months. Nonetheless, the results of the current study indicate that doctors are more likely to experience non-physical WPV. These results may inform interventions aimed at reducing violence affecting healthcare workers by tailoring those interventions to reflect particular violence types, characteristics, and differential risks facing particular healthcare professions.

## 6. Conclusions

The findings from this study have revealed that various occupational groups in hospitals are at risk of WPV in China. More than 80% of the medical staff in seven general hospitals reported exposure to WPV in the previous year. Our findings also revealed that the causes of different types of WPV are not exactly the same. Given our findings, this issue can no longer be ignored, especially from an occupational health and safety perspective. Further study is needed about the deep-seated reasons that violence occurs, in order to reduce the incidence of such violence. Finally, the issue of the strategies about preventing and intervention dealing with workplace violence in hospital should be studied and implemented.

## Figures and Tables

**Table 1:** Participant demographic characteristics.

Characteristic	*n*	%
**Gender**		
Male	742	39.1
Female	1157	60.9
**Age groups (Years of Age)**		
≤29	552	29.1
30–39	765	40.3
≥40	582	30.6
**Years of experience**		
≤5	643	33.9
6–15	696	36.6
≥16	560	29.5
**Marital status**		
Single (never married, divorced, or widowed)	564	29.7
Married	1335	70.3
**Level of education**		
Vocational school	77	4.1
Community college	533	28.0
Undergraduate	959	50.5
Graduate	330	17.4
**Title**		
No title	148	7.8
Primary	626	33.0
Intermediate	612	32.2
Senior	513	27.0
**Occupation**		
Doctors	1086	57.2
Nurses	570	30.0
Medical technology	102	5.4
Administration staff	141	7.4
**Shift work**		
Yes	1347	70.9
No	552	29.1
**Department**		
ICU	39	2.1
Emergency treatment	50	2.6
Internal medicine	705	37.1
Surgery	634	33.4
Gynaecology	121	6.4
Paediatrics	35	1.8
ENT, Department of stomatology	55	2.9
Medical department	119	6.3
Administrative department	141	7.4
**Anxiety level**		
Extremely high	743	39.1
High	278	14.6
Moderate	556	29.3
Low	199	10.5
Never	123	6.5

ICU, intensive care unit; ENT, department of ear nose and throat.

**Table 2:** Violence by occupational category.

Personnel Type	Gender	Total Number	Suffer Physical Violence	Proportion	Suffer Non-Physical Violence	Proportion
Doctors	Male	616	120	19.5%	459	74.5%
	Female	470	54	11.5%	327	69.6%
	Total	1086	174	16.0%	786	72.4%
Nurses	Male	25	5	20.0%	19	76.0%
	Female	545	66	12.1%	386	70.8%
	Total	570	71	12.5%	405	71.1%
Medical technology	Male	43	5	11.6%	24	55.8%
	Female	59	8	13.6%	29	49.2%
	Total	102	13	12.7%	53	52.0%
Administration staff	Male	58	13	22.4%	32	55.2%
	Female	83	3	3.6%	33	39.8%
	Total	141	16	11.3%	65	46.1%
Total		1899	274	14.4%	1309	68.9%

**Table 3:** Characteristics and frequency distributions for physical and non-physical violence.

Characteristics	Total *n*	Physical Violence				Non-Physical Violence *			
		*n*	%	χ^2^	*p*-Value	*n*	%	χ^2^	*p*-Value
**Gender**									
Male	742	143	19.3	23.141	<0.001	534	72.0	5.244	0.022
Female	1157	131	11.3			775	67.0		
**Marital status**									
Single	564	74	13.1	1.112	0.292	400	70.9	1.485	0.223
Married	1335	200	15.0			909	68.1		
**Age groups**									
≤29	552	67	12.1	3.791	0.150	397	71.9	20.601	<0.001
30–39	765	122	15.9			553	72.3		
>39	582	85	14.6			359	61.7		
**Level of education**									
Vocational school	77	8	10.4	16.839	0.001	43	55.8	11.451	0.010
Community college	533	78	14.6			354	66.4		
Undergraduate	959	118	12.3			669	69.8		
Graduate	330	70	21.2			243	73.6		
**Professional title**									
No title	148	18	12.2	6.234	0.101	103	69.6	10.310	0.016
Primary	626	75	12.0			435	69.5		
Intermediate	612	100	16.3			444	72.5		
Advanced	513	81	15.8			327	63.7		
**Shift work**									
Yes	1347	229	17.0	24.830	<0.001	296	53.6	85.149	<0.001
No	552	45	8.2			1013	75.2		
**Occupation**									
Doctors	1086	174	16.0	5.348	0.148	786	72.4	55.252	<0.001
Nurses	570	71	12.5			405	71.1		
Medical technology	102	13	12.7			53	52.0		
Administrative	141	16	11.3			65	46.1		
**Anxiety level**									
Zero	743	9	7.3	87.123	<0.001	54	43.9	85.259	0.000
Low	278	15	7.5			101	50.8		
Moderate	556	40	7.2			387	69.6		
High	199	35	12.6			206	74.1		
Extremely high	123	175	23.6			561	75.5		

* Non-physical violence includes threats, sexual harassment, bullying, and verbal abuse.

**Table 4:** Multiple logistic regression of physical and non-physical violence.

Physical Violence				Non-Physical Violence			
	OR	95% CI	*p*-Value		OR	95% CI	*p*-Value
**Shift work**				**Age groups**			
No	1.0	Reference		≤29	1.359	1.035–1.785	0.027
Yes	1.914	1.338–2.739	<0.001	30–39	1.309	1.025–1.672	0.031
**Anxiety level**				>39	1.0	Reference	
Zero	1.0	Reference		**Shift work**			
Low	0.966	0.405–2.300	0.937	No	1.0	Reference	
Moderate	0.810	0.377–1.739	0.589	Yes	1.894	1.496–2.397	<0.001
High	1.562	0.717–3.401	0.262	**Anxiety level**			
Extremely high	3.229	1.581-6.593	0.001	Zero	1.0	Reference	
**Gender**				Low	1.215	0.763–1.936	0.412
Male	1.860	1.422–2.433	<0.001	Moderate	2.243	1.480–3.398	<0.001
Female	1.0	Reference		High	2.833	1.785–4.499	<0.001
				Extremely high	2.890	1.918–4.355	<0.001
				**Occupation**			
				Doctors	1.609	1.080–2.396	0.019
				Nurses	1.575	1.044–2.374	0.030
				Medical technology	0.909	0.532–1.555	0.728
				Administrative	1.0	Reference	
